# Comparación de las estructuras de difusión de información errónea y verídica en las redes sociales durante una emergencia de salud pública^*^

**DOI:** 10.26633/RPSP.2021.61

**Published:** 2021-05-12

**Authors:** Lida Safarnejad, Qian Xu, Yaorong Ge, Siddharth Krishnan, Arunkumar Bagarvathi, Shi Chen

**Affiliations:** 1 Departamento de software y sistemas de información, Universidad de Carolina del Norte Estados Unidos de América Departamento de software y sistemas de información, Universidad de Carolina del Norte, Estados Unidos de América.; 2 Facultad de Comunicación, Universidad de Elon Estados Unidos de América Facultad de Comunicación, Universidad de Elon, Estados Unidos de América.; 3 Departamento de Ciencias de la Computación, Universidad de Carolina del Norte Estados Unidos de América Departamento de Ciencias de la Computación, Universidad de Carolina del Norte, Estados Unidos de América.; 4 Departamento de Ciencias de la Computación, Universidad Estatal de Oklahoma Estados Unidos de América Departamento de Ciencias de la Computación, Universidad Estatal de Oklahoma, Estados Unidos de América.; 5 Departamento de Salud Pública, Facultad de Ciencias de Datos, Universidad de Carolina del Norte Estados Unidos de América Departamento de Salud Pública, Facultad de Ciencias de Datos, Universidad de Carolina del Norte, Estados Unidos de América.

## Abstract

**Objetivos.:**

Elaborar un esquema operativo integral para detectar la información errónea principal sobre el zika distribuida en Twitter® en el 2016; reconstruir las redes por las que se difunde información mediante retuiteo; contrastar la información verídica frente a la errónea con diversos parámetros; e investigar cómo se difundió en las redes sociales la información errónea sobre el zika durante la epidemia.

**Métodos.:**

Revisamos sistemáticamente los 5 000 tuits más retuiteados con información sobre el zika en inglés, definimos “información errónea” a partir de la evidencia, buscamos tuits que tuvieran información errónea y conformamos un grupo equiparable de tuits con información verídica. Elaboramos un algoritmo para reconstruir las redes de retuiteo de 266 tuits con información errónea y 458 tuits equiparables con información verídica. Calculamos y comparamos nueve parámetros para caracterizar la estructura de las redes a varios niveles, entre los dos grupos.

**Resultados.:**

En los nueve parámetros se aprecian diferencias estadísticamente significativas entre el grupo de información verídica y el de información errónea. La información errónea en general se difunde mediante estructuras más sofisticadas que la información verídica. También hay una considerable variabilidad intragrupal.

**Conclusiones.:**

Las redes de difusión de la información errónea sobre el zika en Twitter fueron sustancialmente diferentes que las de información verídica, lo cual indica que la información errónea se sirve de mecanismos de difusión distintos. Nuestro estudio permitirá formar una comprensión más holística de los desafíos que plantea la información errónea sobre salud en las redes sociales.

## Abstract (en)

**Objectives.:**

To provide a comprehensive workflow to identify top influential health misinformation about Zika on Twitter in 2016, reconstruct information dissemination networks of retweeting, contrast mis- from real information on various metrics, and investigate how Zika misinformation proliferated on social media during the Zika epidemic.

**Methods.:**

We systematically reviewed the top 5000 English-language Zika tweets, established an evidence-based definition of “misinformation,” identified misinformation tweets, and matched a comparable group of real-information tweets. We developed an algorithm to reconstruct retweeting networks for 266 misinformation and 458 comparable real-information tweets. We computed and compared 9 network metrics characterizing network structure across various levels between the 2 groups.

**Results.:**

There were statistically significant differences in all 9 network metrics between real and misinformation groups. Misinformation network structures were generally more sophisticated than those in the real-information group. There was substantial within-group variability, too.

**Conclusions.:**

Dissemination networks of Zika misinformation differed substantially from real information on Twitter, indicating that misinformation utilized distinct dissemination mechanisms from real information. Our study will lead to a more holistic understanding of health misinformation challenges on social media.

Las redes sociales se han convertido en fuentes de información en tiempo real sobre diversos temas, incluidas la medicina y la salud (1, 2). En una red social como Twitter, los contenidos los generan principalmente los usuarios; al no haber mecanismos eficaces de verificación, las redes sociales pueden ser un medio para propagar información errónea. La infiltración y proliferación de la información errónea sobre la salud en las redes sociales, especialmente durante las emergencias de salud pública, constituye una amenaza grave para las personas y para la sociedad en su conjunto (3). Algunos ejemplos de este problema es la información errónea difundida en relación con las vacunas (4-6), el zika (7), el tabaco, el vapeo o los productos de marihuana (8).

Si bien las redes sociales pueden ser una herramienta eficaz para mejorar la alfabetización en la salud (9, 10), también son un recurso rico para estudiar las perspectivas y reacciones del público ante diversos temas (11-14). Los sistemas de infovigilancia tienen como objetivo fortalecer la capacidad de la comunidad de salud pública, haciendo un seguimiento meticuloso de los debates en línea sobre temas de salud (15-18) y detectando la información errónea (19). Los análisis más modernos de la difusión de información (errónea o no) en las redes sociales persiguen dos grandes propósitos: 1) analizar la cascada de información (20, 21); y 2) detectar la información errónea (22-26). En la primera dirección, se utilizan modelos computacionales para investigar la viralidad y la difusión de información; en la segunda, se examinan diferentes atributos, principalmente de contexto, para detectar la información errónea (23, 24, 27-33). Sin embargo, resulta difícil elaborar este tipo de sistemas a partir del contenido textual (34), ya que es posible alterar dicho contenido para que parezca verídico y escape a la detección de los algoritmos automatizados (35). Nosotros planteamos que el contenido textual es solo un aspecto del problema general que supone la información errónea sobre la salud en las redes sociales, por lo que no es adecuado confiar únicamente en él. Urge comprender el problema de la información errónea sobre la salud desde más aspectos, además del contenido, como los usuarios involucrados y las redes sociales como entorno interconectado.

Una de las actuaciones decisivas para investigar un brote epidémico es rastrear su trayectoria. En este estudio, definimos la difusión de información errónea como un proceso dinámico por el cual la publicación original (p. ej., un tuit) se propaga mediante retuiteo por una red de difusión de información (denominada llanamente “red” en lo sucesivo). El retuiteo indica que el usuario reconoce la importancia de la publicación original y está dispuesto a difundir la información. Por lo tanto, analizamos el retuiteo como método de difusión (35).

Como caso de estudio tomamos la epidemia del zika del 2016. En aquel entonces, los organismos de salud potenciaron su presencia en las redes sociales a fin de transmitir los descubrimientos y las orientaciones más recientes. Sin embargo, la incertidumbre sobre la epidemia y algunos eventos paralelos ocurridos ese mismo año, como las elecciones presidenciales en Estados Unidos y los Juegos Olímpicos de Río de Janeiro, abrieron la puerta a la información errónea. En este estudio, primero establecimos una definición operativa de información errónea sobre salud y buscamos los tuits más populares que contuviesen información errónea sobre el zika. A continuación, elaboramos un algoritmo para inferir y reconstruir las redes de difusión de los principales mensajes erróneos sobre el zika y de tuits equiparables con información verídica. Después, aplicamos análisis reticulares para extraer parámetros relativos a la estructura de la red en ambos grupos. Investigamos cómo las estructuras reticulares difieren cuantitativamente. Este estudio mejora nuestro conocimiento sobre el mecanismo de difusión por las redes sociales de la información errónea de salud, y sobre su mayor eficiencia para difundirse en comparación con la información verídica. Además, lo descubierto con este proyecto ayudará a formular estrategias de comunicación en salud más eficaces para combatir la información errónea en las redes sociales.

## MÉTODOS

Como período de muestreo elegimos el año 2016 completo (del 1 de enero al 31 de diciembre). Este período abarca los principales sucesos en el cronograma de la epidemia de zika: la advertencia inicial de la Organización Mundial de la Salud (OMS) en la Región de las Américas, la declaración oficial de la emergencia de salud pública de importancia internacional y el fin de dicha emergencia. Tomando “zika” como palabra clave, recopilamos un total de 3,7 millones de tuits y retuits en inglés, publicados en el 2016, mediante la interfaz de programación de aplicaciones Gnip, a través del programa de ciencias de datos de nuestra universidad. Este conjunto de datos completo está integrado, así, por todas las conversaciones sobre el zika, en inglés, mantenidas en Twitter en el 2016. Por lo tanto, nuestro conjunto ofrece una visión más completa e imparcial del discurso público sobre el zika que tuvo lugar en Twitter a lo largo de ese año.

### Detección de información errónea y equiparación con información verídica

Clasificamos todos los tuits originales sobre el zika según el número de veces que fueron retuiteados, de mayor a menor. Después de esta clasificación descendente, seleccionamos los 5000 tuits más retuiteados como muestra. A continuación, establecimos una definición operativa de “información errónea”, es decir, que la información contenida en el tuit no estuviese basada en evidencia. Para evaluar la validez de un tuit, utilizamos artículos de revistas especializadas y actas de congresos con revisión externa, informes y estadísticas de organismos oficiales y de salud (p. ej., los Centros para el Control y la Prevención de Enfermedades y la OMS) y sitios web de verificación de datos. Después de detectar la información errónea en el grupo de 5000 tuits más difundidos, configuramos otro grupo de tuits equiparables, que contenían información verídica, teniendo en cuenta el momento de publicación y las veces que fueron retuiteados. El apéndice A contiene la descripción pormenorizada de cómo se aplicó esta definición, la garantía de fiabilidad y algunos ejemplos (suplemento en inglés a la versión en línea de este artículo en http://www.ajph.org).

Adquirimos los metadatos de cada tuit original y de todos sus retuits, a saber: fecha y hora de publicación, nombre de los usuarios involucrados e información sobre seguidores y seguidos. Utilizamos estos metadatos para rastrear la difusión de la información, construir las redes y realizar los análisis pertinentes.

### Construcción de las redes dinámicas de difusión de información

Como Twitter no publica explícitamente quién retuitea a quién, para inferir y reconstruir la red de retuiteos elaboramos un algoritmo basado en el momento de publicación o retuiteo y la relación entre amigos y seguidores. En el apéndice B se brinda un informe técnico detallado sobre este algoritmo (suplemento en inglés a la versión en línea de este artículo en http://www.ajph.org).

También investigamos la dinámica cronológica de la difusión de la información, calculando el tiempo que se tarda en conseguir el 50%, 75%, 90%, 95% y 100% de todos los retuits en el grupo de información verídica y el grupo de información errónea. Con este cálculo se constata la variabilidad cronológica dentro de cada grupo y entre los dos grupos, además de conferir a las redes una dimensión temporal.

### Computación e interpretación de los parámetros relativos a las redes

Una vez construida la red de cada tuit, calculamos y comparamos los parámetros que resultan más relevantes para la difusión de información, dentro de cada grupo y entre los dos grupos. En este estudio, calculamos nueve atributos: alcance de la red (ALC), influencia de la red (INF), diámetro (DIA), densidad (DEN), modularidad (MOD), índice de Wiener (WIE), viralidad estructural (VIR), puntuación máxima de centralidad de grado de salida (SAL) y puntuación máxima de centralidad de intermediación (INT). Con estas mediciones se cuantificó y caracterizó la estructura de las redes, desde diferentes aspectos y a diferentes niveles dentro de la red. A continuación, describimos sucintamente estas medidas; en el apéndice C se brindan explicaciones técnicas más detalladas (suplemento en inglés a la versión en línea de este artículo en http://www.ajph.org):

- ALC: mide el número de nodos únicos (es decir, perfiles de usuario individuales dentro de la red).
- INF (también denominada “tamaño de la red”): representa el número de todos los nodos. Si cada usuario retuitea exactamente una vez, ALC debe ser igual a INF. Si la diferencia entre ALC e INF es mayor, significa que algunos usuarios de la red retuitearon más de una vez.
- DIA: distancia más corta entre los dos nodos más alejados dentro de la red.
- VIR: distancia media entre todos los pares de nodos de la red.
- DEN: proporción de relaciones potenciales que realmente existen en la red.
- WIE: suma de las trayectorias más cortas entre todos los pares de nodos.
- MOD: probabilidad de dividir una red en posibles conglomerados (es decir, subgrupos); dentro de un mismo subgrupo, hay gran interconexión de los nodos, pero entre un subgrupo y otro la interconexión es más débil. La MOD es una medición reticular de nivel local.
- SAL: mide la influencia que tiene un solo nodo al generar retuits que difunden información. Calculamos toda la distribución de SAL y presentamos el valor más alto de todos los perfiles que retuitean dentro de una misma red.
- INT: cuantifica la importancia del nodo en lo referente a la conectividad de la red. Cuanto mayor es INT, más importante es el nodo para mantener la estabilidad de la red. Exponemos el valor de INT más alto.

En resumen, con estos parámetros se caracterizaron detalladamente los diferentes aspectos de las redes a múltiples escalas, desde el nivel de red global (ALC, INF, DIA, VIR, DEN y WIE), pasando por el conglomerado local (MOD), hasta el nodo individual (SAL e INT). Aunque hay otras mediciones, estas nueve son especialmente críticas para la difusión de información desde el perfil de usuario que publica el tuit original a otros nodos de retuiteo dentro de la red.

### Análisis reticulares y análisis estadísticos

Comparamos las mediciones reticulares entre los dos grupos mediante la prueba de Kolmogorov-Smirnov para detectar diferencias significativas en las distribuciones. Los datos los recuperamos, procesamos y reconstruimos con Python, versión 3.7.3® (Python Software Foundation, Beaverton, Oregón, Estados Unidos). Realizamos los análisis reticulares y estadísticos con R, versión 3.5.0® (R Foundation, Viena), con paquetes adicionales. Si se desean consultar, se pueden solicitar gratuitamente todos los datos y códigos de entrada.

## RESULTADOS

En este estudio, prestamos atención a los tuits sobre el zika más populares. Definimos la popularidad como el número de veces que se retuitea un tuit. Consideramos que un tuit era popular si se retuiteaba al menos 50 veces. En nuestra muestra hay cerca de 5 000 tuits que superan esa cifra. De los 5 000 tuits sobre el zika más retuiteados en el 2016, detectamos un total de 400 que contenían información errónea y los verificamos. De ellos, 266 contenían suficientes metadatos para reconstruir la red de difusión. No todos los metadatos estaban disponibles: algunos se habían perdido porque Twitter había prohibido algunos perfiles o borrado algunos contenidos, o porque el usuario se había retractado de la publicación original, por diversas razones. El grupo de comparación (información verídica) está integrado por 458 tuits, publicados sobre las mismas fechas que los tuits con información errónea y retuiteados una cantidad de veces semejante. Para evitar un posible sesgo de selección, no hicimos una equiparación de los tuits verídicos uno a uno.

### Variabilidad cronológica de la dinámica de difusión de información

**FIGURA 1.**
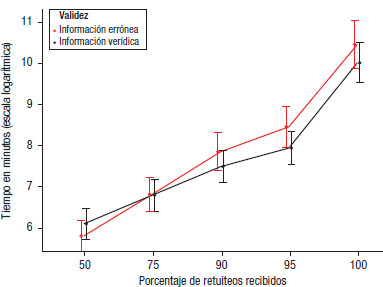
Heterogeneidad cronológica del retuiteo en el grupo de información verídica y el de información errónea sobre el zika (2016)

Se observa una variabilidad cronológica sustancial en la dinámica de retuiteo entre el grupo de información errónea y el grupo información verídica (figura 1). La información errónea tardó significativamente menos tiempo en conseguir el 50% de todos los retuits (*T*_50_ = 334 min, información errónea; *T*_50_ = 448 min, información verídica; *P* < 0,001, prueba bilateral de la *t*). La diferencia es mínima cuando se tiene en cuenta el 75% de todos los retuits (*T*_75_ = 916 min, información errónea; *T*_75_ = 898 min, información verídica; *P* = 0,93). Curiosamente, a partir de ese momento, la información errónea siempre tarda significativamente más tiempo en conseguir el 90% de los retuits (*T*_90_ = 2 580 min, frente a *T*_90_ = 1 795 min; *P* = 0,03), 95% de los retuits (*T*_95_ = 4 739 min; frente a *T*_95_ = 2 824 min; *P* = 0,001) y todos los retuits (*T*_100_ = 34 869 min, frente a *T*_100_ = 22 340 min; *P* < 0,001). La información errónea consiguió al menos la mitad de todos los retuits en un período relativamente corto, para viralizarse más. Posteriormente, es posible que se la retuitee deliberadamente para mantener su visibilidad durante un período más prolongado. A partir de estas observaciones, elegimos el tiempo transcurrido hasta el último retuit para construir la red, ya que proporciona la visión más completa de la actividad de retuiteo.

*Nota:* El eje y (tiempo) está en escala logarítmica natural. El eje x (porcentaje de retuiteos) no está posicionado por cifras absolutas. Por término medio, la información errónea (en rojo) tarda mucho menos en conseguir el 50% de todos los retuiteos, en comparación con la información verídica (en negro), pero tarda significativamente más tiempo en lograr el 90% y porcentajes más altos.

### Comparación de las mediciones reticulares entre los dos grupos

**FIGURA 2.**
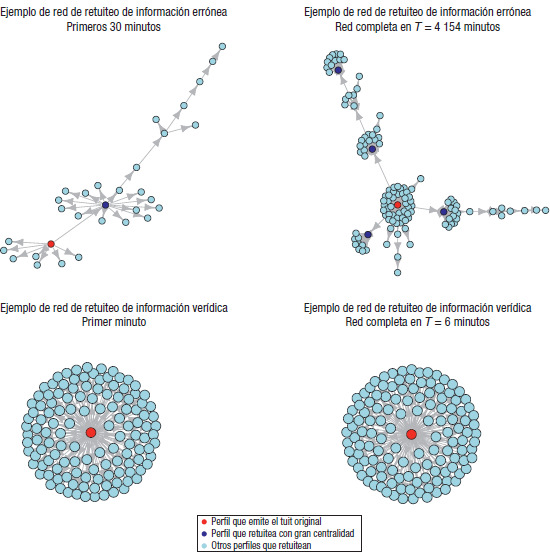
Ejemplos de redes de retuiteo de información errónea y verídica sobre el zika (2016)

Reconstruimos las redes de retuiteo de cada tuit dentro del grupo de información verídica y el de información errónea. En la figura 2 se brindan ejemplos de estructuras reticulares dinámicas, tanto de la información errónea como de la verídica.

*Nota:* Estas redes ilustran cómo se infiltran la información verídica y errónea en los perfiles que retuitean. La contribución relativa del perfil puede cuantificarse por su puntuación de centralidad. Obsérvese que, en este ejemplo, es posible que la información verídica hay sido difundida por bots. Por lo tanto, los bots no necesariamente se asocian solo con la información errónea.

En el cuadro 1 se recogen las distribuciones de las mediciones reticulares importantes en ambos grupos. A efectos de la demostración, transformamos todas las mediciones a una escala de 0 a 1, mediante el escalado de atributos. En el cuadro 1 se recogen las estadísticas numéricas reales. Ninguna de estas distribuciones se aproxima a la normalidad, lo cual indica que tienen un alto índice de asimetría y curtosis, así como posible multimodalidad. Esto es señal de gran variabilidad intragrupal en las estructuras de las redes.

Todas las distribuciones de las mediciones reticulares difieren significativamente (*P* < 0,05) entre el grupo de información verídica y el de información errónea, según la prueba de Kolmogorov-Smirnov. Por lo tanto, las redes de información verídica y errónea presentan muchas heterogeneidades, tanto intragrupales como intergrupales.

La DEN es significativamente mayor en el grupo de información errónea, ya que los usuarios que retuitean tienden a retuitear información errónea si la tuitean o retuitean sus amigos (o los usuarios a los que siguen). Téngase en cuenta que esta es la red original de seguidores y seguidos a partir de la cual se infirió la red de difusión de información. En el apéndice B se detalla la diferencia y la relación entre estas dos redes. En el apéndice C se explica el motivo por el que usamos la red original solo para determinar la densidad.

**CUADRO 1.** Estadísticas relativas a las mediciones reticulares en el grupo de información errónea y el grupo de información verídica sobre el zika en Twitter (2016)

Medición	DIA	WIE	DEN	TAM	ALC	VIR	MOD	INT	SAL
Información errónea Mínimo	1	1	0	0	1	0,5	−0,5	0	1
25%	8	4,10 × 10^4^	3,1 × 10^−3^	135	136	3,53	0,47	66	43
50%	15	1,14 × 10^5^	5,2 × 10^−3^	263	264	6,49	0,68	278	89
75%	25	3,95 × 10^5^	8,5 × 10^−3^	321	322	10,23	0,79	881	148
Máximo	259	5,19 × 10^6^	2,5 × 10^−1^	1 456	1 457	60,82	0,93	35 451	844
Media	20	8,84 × 10^5^	9,0 × 10^−3^	264	263	8,01	0,61	1 003	115
EE	5	2,26 × 10^4^	2,0 × 10^−3^	14	13	0,4	0,01	170	7
IC95%	10 - 30	4,42 × 10^5^ - 1,33 × 10^6^	5,1 × 10^−3^ - 1,3 × 10^−2^	237 - 291	238 - 288	7,23 - 8,79	0,59 - 0,63	670 – 1 336	101 - 448
Información verídica Mínimo	1	1	0	0	1	0,5	−0,5	0	1
25%	3	1,54 × 10^4^	5,5 × 10^−3^	111	112	2,19	0,18	4	57
50%	5	2,71 × 10^4^	6,9 × 10^−3^	141	145	2,62	0,37	15	97
75%	8	6,02 × 10^4^	8,8 × 10^−3^	178	179	4,09	0,6	110	131
Máximo	92	1,31 × 10^5^	2,5 × 10^−1^	341	342	32,83	0,88	3 567	250
Media	8	5,80 × 10^4^	1,0 × 10^−2^	145	144	3.82	0,39	127	97
EE	0,4	4,83 × 10^3^	1,0 × 10^−3^	3	3	0,15	0,01	15	2
IC95%	7 - 9	4,85 × 10^4^ - 6,74 × 10^4^	8,0 × 10^−3^ - 1,2 × 10^−2^	139 - 151	138 - 150	3,53 - 4,11	0,37 - 0,41	98 - 156	93 - 101

ALC: alcance; DEN: densidad de la red de seguidores-seguidos (no de la red de difusión de información; véase el apéndice C, que contiene una explicación detallada, como suplemento a la versión en línea de este artículo, en http://www.ajph.org); DIA: diámetro; EE: error estándar; IC: intervalo de confianza; INT: puntuación máxima de centralidad de intermediación de un nodo; MOD: modularidad; SAL: puntuación máxima de centralidad de grado de un nodo; TAM: tamaño; VIR: viralidad; WIE: índice de Wiener. Se utilizó la prueba bilateral de Kolmogorov-Smirnov para investigar si la distribución de determinadas mediciones era significativamente diferente entre los grupos (es decir, si las medidas seguían distribuciones diferentes). En las nueve mediciones se aprecian diferencias estadísticamente significativas (*P* < 0,001) entre el grupo de información verídica y el de información errónea.

El DIA también es significativamente mayor en el grupo de información errónea. En general, cuanto menor es el diámetro, menos capas tiene que atravesar la información, en sentido periférico, hasta llegar al último usuario que la retuitea. La información errónea atrae a más usuarios de base, de forma consecutiva, mientras que la información verídica sigue una difusión más jerárquica, en forma de cascada.

La VIR, con la que se mide la longitud media de la trayectoria, también es significativamente más alta en el grupo de información errónea, lo cual indica que, en general, los nodos están más separados dentro la red. Este dato refuerza nuestra observación anterior de que la información errónea se difunde de usuario a usuario de forma más directa, o entre pequeños conglomerados, mientras que la información verídica sigue una difusión jerárquica, en capas.

La INF y el ALC son mediciones análogas, por cuanto el ALC se utilizó para medir usuarios individuales que retuitean. El ALC y la INF son significativamente más pequeños en el grupo de información errónea. Además, alrededor del 30% de los tuits con información errónea tenían un mismo nodo retuiteado al menos dos veces, cifra sustancialmente más alta que en el grupo de información verídica (<10%). Esto podría consistir en una estrategia de propagación intencional para difundir información errónea en las redes sociales. Sin embargo, el riesgo de tal estrategia es que Twitter la detecte y actúe. Por lo tanto, que varios perfiles retuiteen el mismo contenido juntos sería una forma más eficaz de difundir información errónea que retuitear el mismo contenido varias veces desde un mismo perfil.

Respecto al WIE, el grupo de información errónea presenta, por término medio, valores significativamente más bajos. Esto indica que las redes de retuiteo de información errónea pueden tener conglomerados locales con más forma de estrella, lo cual reduce el WIE. También concuerda con lo que ya habíamos observado antes: que la red de información errónea tiene más conglomerados locales y un valor MOD más alto. Sin embargo, este hallazgo parece contradecir la constatación anterior de que, por término medio, el DIA es en realidad más alto en el grupo de información errónea. La distribución real del WIE en el grupo de información errónea es la clave para resolver este dilema (cuadro 1). En el grupo de información errónea, la distribución de WIE tiene más de un pico prominente (es decir, es multimodal). Si bien algunas redes de información errónea sobre el zika tienen valores de WIE más bajos, otras tienen WIE muy altos. Desde la perspectiva real de propagación de la información errónea, esto significa que los propagadores explotan dos estrategias aparentemente divergentes: en primer lugar, utilizan una red asteriforme, con un WIE muy bajo (mucho menos frecuente en el grupo de información verídica); en segundo lugar, utilizan una red de difusión semejante a una cadena, con un WIE alto. Además, hay híbridos de estas estrategias para difundir la información errónea más lejos. Por ejemplo, algunos propagadores crean una ráfaga inicial de retuits, que aparecen como estrellas locales en la red, lo que atrae a más usuarios de base que adoptan la tendencia y retuitean uno tras otro. En cambio, no observamos una ordenación tan sofisticada en el grupo de información verídica.

A nivel de la red local, observamos una mayor modularidad en el grupo de información errónea, lo cual indica que los usuarios que la retuitearon formaron conglomerados locales más pequeños para difundirla. Por lo tanto, es más difícil atajar la información errónea sobre el zika con los métodos tradicionales de mitigación. Cuando hay varios conglomerados de menor tamaño, el riesgo de que desaparezcan algunos es menor, ya que otros conglomerados sirven como ruta alternativa. En comparación, el grupo de información verídica tiene una MOD más baja. En el grupo de información errónea, la MOD está más escorada hacia la izquierda que en el grupo de información verídica (cuadro 1).

A nivel de nodo, la distribución de SAL en el grupo de información errónea también tiene un patrón marcadamente multimodal, lo que indica que en muchos tuits con información errónea interviene un usuario con un grado de salida muy alto (puntuación de centralidad >200; cuadro 1), que puede actuar como perfil influyente o propagador en línea. Por otro lado, la distribución de SAL en el grupo de información verídica se asemeja a una distribución normal. En el grupo de información errónea, el SAL está más escorado hacia la derecha que en el grupo de información verídica. La inmensa mayoría de los usuarios del grupo de información errónea no influyeron en la transmisión de la información.

Respecto a la INT, los usuarios del grupo de información verídica con INT más altas tienen una puntuación significativamente más baja que en el grupo de información errónea (127 frente a 1.003). Por lo tanto, estos usuarios resultan más importantes en el grupo de información errónea que en el de información verídica para mantener la estabilidad de la red, ya que una INT alta indica un papel más relevante en la transmisión de información. Si bien es relativamente fácil identificar a los usuarios con mayor SAL, por su actividad superficial de atracción de gran cantidad de retuits, es mucho más difícil de detectar el usuario con INT más alta, a menos que se construya la red y se realicen cálculos de centralidad en relación con todos los nodos. No obstante, desde una perspectiva de mitigación de la información errónea, dirigirse a los usuarios con INT más altas puede ser una manera más eficaz de frenar e incluso desarticular por completo la propagación, en vez de actuar solo sobre los usuarios con SAL más altas.

En resumen, el grupo de información errónea presenta distribuciones distintas de todas las mediciones reseñadas, lo cual indica que sus redes de difusión tienen estructuras significativamente diferentes que las de la información verídica. La extracción de datos sobre las redes de difusión puede ayudar a los profesionales de la salud y al público general a comprender mejor cómo se difunde la información errónea sobre temas de salud. Además, se pueden usar estos indicadores cuantitativos en informática de la salud a fin de diseñar sistemas más precisos de infovigilancia y detección de información errónea.

## DISCUSIÓN

Hemos elaborado un marco analítico para investigar la difusión de la información errónea sobre salud en las redes sociales. Aportamos una definición operativa de “información errónea sobre la salud” y construimos un algoritmo para rastrear explícitamente cómo se difunde dicha información en las redes sociales, siguiendo redes de retuiteo.

Es preciso señalar que nuestro conocimiento actual sobre temas de salud evoluciona a medida que disponemos de más evidencia clínica, epidemiológica y de otra índole: de ahí el concepto de “basado en la evidencia”. Los términos “información verídica” e “información errónea” deben emplearse con precaución, porque nuestras nociones actuales pueden demostrarse falsas en el futuro. Conviene tener en cuenta el momento en el que se produce la conversación, especialmente durante una crisis de salud emergente, como fue la epidemia del zika. Por ejemplo, observamos que *The Economist*, considerado una fuente fidedigna, tuiteó en diciembre del 2016 que el zika era inofensivo para los adultos, cuando ya había evidencia que demostraba claramente la relación causal entre la infección por el virus del zika y el síndrome de Guillain-Barré en adultos (posteriormente eliminaron ese tuit). Si el tuit hubiera aparecido a principios del 2016, cuando la relación causal aún no estaba probada, no se habría considerado información errónea. Como consecuencia, un aspecto importante que se desprende de este estudio es la necesidad de incrementar la alfabetización sobre la salud de la sociedad, de manera que las personas sepan comprobar la validez de la información y determinar cuándo es errónea, y actualicen con frecuencia sus conocimientos sobre los problemas de salud, en lugar de decir sencillamente que una información es verdadera o no.

Seguiremos investigando la actividad y los atributos de los usuarios para detectar *bots* y examinar su eficacia para difundir información verídica y errónea en las redes sociales. Sospechamos que la difusión de información verídica que mostramos como ejemplo (figura 2) fue facilitada por *bots*. Además, comprobamos la verificación de los usuarios y descubrimos que la proporción de usuarios verificados era pequeña. En el grupo de información verídica, el porcentaje de usuarios verificados es más alto que en el grupo de información errónea (2% frente al 1,2%). Esta observación coincide con otros estudios, en los que se aprecia una fuerte presencia en Twitter de agencias de noticias acreditadas y organismos de salud durante el brote del zika del 2016.

En este momento están realizándose otros estudios para investigar cómo se comportan los usuarios a lo largo del tiempo y cómo esa dinámica temporal revela la infiltración de información errónea. En este estudio, construimos la red *G* de un determinado tuit una vez concluido todo el retuiteo. La gran heterogeneidad cronológica observada, tanto intragrupal como intergrupal (figura 1), evidencia la capacidad de nuestro algoritmo de construir la red dinámica *G_t_* en un momento dado *t*. Si detectamos un aumento repentino del retuiteo en el momento *t*, podemos construir una red específica del momento *t,* para determinar explícitamente qué usuario está causando la explosión de retuiteos, cuantificar las puntuaciones de centralidad del usuario y trabajar a nivel de nodo para abordar en mayor profundidad la epidemia de información errónea sobre la salud en las redes sociales.

### Implicaciones para la salud pública

Este estudio proporciona evidencia sólida sobre los mecanismos de difusión de la información errónea sobre la salud en Twitter, una de las redes sociales más utilizadas. Nuestro marco analítico posee un desarrollo universal y sirve para explorar otros problemas de salud pública en las redes sociales. Por ejemplo, hemos estudiado la información errónea que circula sobre los organismos genéticamente modificados en Sina Weibo, la mayor red social de China. Con este marco también se están investigando otras cuestiones candentes que suscitan polémica, como la actual pandemia de COVID–19.

Otra implicación fundamental para la salud pública que tiene nuestro estudio es la posibilidad de conocer algunos atributos importantes de la información errónea de salud, imposibles de identificar directamente solo por su contenido. Hemos demostrado la importancia de tratar la información errónea (patógeno), los usuarios (huéspedes) y las redes sociales (entorno) como una entidad interconectada: la tríada infodemiológica. La información errónea, al igual que los patógenos reales, no circula sin dejar rastro. Este estudio nos ha permitido conocer mucho mejor las dinámicas de difusión de la información errónea. Además, el nutrido conjunto de datos puede servir, junto con otras características (p. ej., contenido, aspectos lingüísticos y datos de los perfiles), para crear un detector integral de información errónea sobre temas de salud. En trabajos posteriores, utilizaremos los métodos más avanzados de aprendizaje automático para crear un clasificador de este tipo, destinado a aplicaciones de salud pública.

### Conclusiones

Hemos investigado cómo se difunde la información verídica y errónea por las redes sociales durante una emergencia de salud, desde la perspectiva de redes dinámicas. Descubrimos que los dos grupos siguen estructuras distintas, lo que indica que poseen diferentes patrones de difusión. Nuestro trabajo arroja luz sobre el desarrollo de detectores más precisos de información errónea.

## Declaración

Las opiniones expresadas en este manuscrito son responsabilidad del autor y no reflejan necesariamente los criterios ni la política de la *RPSP/PAJPH* y/o de la OPS.
